# Prevalence and correlates of suicidal behaviours in a representative epidemiological youth sample in Hong Kong: the significance of suicide-related rumination, family functioning, and ongoing population-level stressors

**DOI:** 10.1017/S0033291722001519

**Published:** 2023-07

**Authors:** Stephanie M. Y. Wong, Charlie H. Ip, Christy L. M. Hui, Y. N. Suen, Corine S. M. Wong, W. C. Chang, Sherry K. W. Chan, Edwin H. M. Lee, Simon S. Y. Lui, K. T. Chan, Michael T. H. Wong, Eric Y. H. Chen

**Affiliations:** 1 Department of Psychiatry, School of Clinical Medicine, LKS Faculty of Medicine, The University of Hong Kong, Hong Kong; 2 The State Key Laboratory of Brain and Cognitive Sciences, The University of Hong Kong, Hong Kong

**Keywords:** COVID-19, epidemiological study, family functioning, risk factors, suicidal behaviours, suicide-related rumination, youth mental health

## Abstract

**Background:**

Young people are most vulnerable to suicidal behaviours but least likely to seek help. A more elaborate study of the intrinsic and extrinsic correlates of suicidal ideation and behaviours particularly amid ongoing population-level stressors and the identification of less stigmatising markers in representative youth populations is essential.

**Methods:**

Participants (*n* = 2540, aged 15–25) were consecutively recruited from an ongoing large-scale household-based epidemiological youth mental health study in Hong Kong between September 2019 and 2021. Lifetime and 12-month prevalence of suicidal ideation, plan, and attempt were assessed, alongside suicide-related rumination, hopelessness and neuroticism, personal and population-level stressors, family functioning, cognitive ability, lifetime non-suicidal self-harm, 12-month major depressive disorder (MDD), and alcohol use.

**Results:**

The 12-month prevalence of suicidal ideation, ideation-only (no plan or attempt), plan, and attempt was 20.0, 15.4, 4.6, and 1.3%, respectively. Importantly, multivariable logistic regression findings revealed that suicide-related rumination was the only factor associated with all four suicidal outcomes (all *p* < 0.01). Among those with suicidal ideation (two-stage approach), intrinsic factors, including suicide-related rumination, poorer cognitive ability, and 12-month MDE, were specifically associated with suicide plan, while extrinsic factors, including coronavirus disease 2019 (COVID-19) stressors, poorer family functioning, and personal life stressors, as well as non-suicidal self-harm, were specifically associated with suicide attempt.

**Conclusions:**

Suicide-related rumination, population-level COVID-19 stressors, and poorer family functioning may be important less-stigmatising markers for youth suicidal risks. The respective roles played by not only intrinsic but also extrinsic factors in suicide plan and attempt using a two-stage approach should be considered in future preventative intervention work.

## Introduction

Large-scale population-level crises, such as the coronavirus disease 2019 (COVID-19) and social unrest, are increasingly common in today's global society (Marani, Katul, Pan, & Parolari, 2021; Ni et al., 2020). Apart from their consequences on mental health (Torales, O'Higgins, Castaldelli-Maia, & Ventriglio, 2020; Wong et al., 2021a), concerns have been raised regarding their impacts on suicidal risks (Gunnell et al., 2020). Most existing studies, however, tended to focus on intrinsic vulnerabilities and personal life stressors and rarely accounted for the influences of ongoing societal circumstances. Notably, suicide remains to be one of the leading causes of mortality and disability, with young people being particularly vulnerable to suicidal thoughts and behaviours (O'Connor & Nock, 2014; World Health Organization, 2021). Self-disclosure of suicidal behaviours and help-seeking are, nonetheless, disproportionately suboptimal due to stigma (Calear, Batterham, & Christensen, 2014; Sheehan, Corrigan, & Al-Khouja, 2017). With the persisting challenges in predicting suicidal risks (Turecki & Brent, 2016), the identification of novel markers that are less stigmatising is crucial for facilitating early risk detection and intervention.

Suicidal ideation, plan, and attempt are among the three most commonly studied non-fatal suicidal behaviours that vary on a continuum of severity (Nock et al., 2013; Turecki & Brent, 2016). While suicidal ideation is more common (Nock et al., 2008), this milder form of suicidal behaviour is often considered to precede suicide planning and attempt (Hintikka et al., 2009). Each of these outcomes thus represents important intervention targets (Turecki & Brent, 2016). Few studies, however, have examined how a range of personal and environmental risk factors may be similarly or differentially associated with all three outcomes in epidemiological youth samples.

Various individual-level risk factors for suicidal behaviours have been discussed, including younger age, higher levels of hopelessness and neuroticism, self-harm, as well as pre-existing psychiatric conditions, particularly depressive disorder and excessive substance use (Cha et al., 2018; Mars et al., 2019). Cognitive deficits, such as updating, have also been suggested to be associated with greater suicidal risks (Bredemeier & Miller, 2015). These factors are, nonetheless, either difficult to modify or are non-specific to suicidal outcomes. While self-harm and psychiatric conditions may be strong indicators of risk, young people are typically also less willing to disclose such experiences and seek help (Gulliver, Griffiths, & Christensen, 2010; Michelmore & Hindley, 2012; Rowe et al., 2014).

One potentially important yet seldomly investigated risk factor is *suicide-related rumination*. Prior studies that examined rumination in mental health and suicidal outcomes have typically emphasised the “self-focused” depressive-type rumination, in which the thought content surrounds one's distress symptoms and their causes and consequences (Miranda & Nolen-Hoeksema, 2007; Rogers & Joiner, 2017). Suicide-related rumination, in contrast, describes repetitive thoughts *about* suicide, which may hold greater relevance to the determination of suicidal risks. Indeed, a few initial studies have offered support for suicide-related rumination as an indicator of suicidal risks (Höller, Teismann, & Forkmann, 2021; Rogers & Joiner, 2018; Rogers, Gallyer, & Joiner, 2021). Notably, a recent study has found depressive rumination to be no longer predictive of lifetime ideation and attempt in both student and community adult samples when suicide-related rumination was accounted for (Rogers, Law, et al., 2021), highlighting its potential relevance.

In addition, considering the roles of both proximal and distal environmental stressors can be important. For instance, the impact of exposure to personal stressful life events (SLEs) on suicide has generally been studied (Stewart et al., 2019; Turecki and Brent, 2016). Family functioning has also been raised to be a key factor of youth suicide (Law & Shek, 2016; Leung, Kwok, & Ling, 2016), which is particularly relevant to Hong Kong since most young people tend to live with their family due to limited living space and high cost of living. How population-level environmental stressors, such as social unrest and COVID-19 – which have been associated with increased mental health risks (Ni et al., 2020; Wong et al., 2021b) – may additionally contribute to different suicidal outcomes in epidemiological samples remains to be studied.

Findings from a limited number of studies on this topic have suggested the influence of epidemics on suicidal behaviours (Leaune, Samuel, Oh, Poulet, & Brunelin, 2020; Zortea et al., 2021). While mixed findings have been reported concerning suicidal risks before and during COVID-19 (Cheung, Lam, Lee, Xiang, & Yip, 2021; Sher, 2020), a recent study has found disproportionately higher rates of suicidal ideation during COVID-19 among young adults in Hong Kong compared to other populations (Schluter et al., 2021). This finding was partly attributed by the researchers to the cumulative effects of the series of social unrest since June 2019 (which had only gradually subsided since the local COVID-19 pandemic in January 2020) and the pandemic (Schluter et al., 2021). Indeed, the additive effects of social unrest-related traumatic events (TEs) and COVID-19 pandemic-related events (PEs) on psychopathological symptoms in young people have previously been reported (Wong et al., 2021a).

The current study thus aimed to first provide an update on the prevalence of lifetime and 12-month suicidal ideation, plan, and attempt in a large representative epidemiological youth sample in Hong Kong. We also aimed to examine factors associated with these three key suicide-related outcomes using a comprehensive multifactorial model, covering aspects ranging from demographics to psychological and personality factors, interpersonal relationships, psychiatric conditions, as well as suicide-related rumination, cognitive ability, and both proximal and distal personal and population-level stressors.

## Method

### Population and study design

Participants were consecutively recruited between September 2019 and 2021 as part of the ongoing Hong Kong Youth Epidemiological Study of Mental Health (HK-YES). The HK-YES is an ongoing territory-wide, household-based epidemiological study of youth mental health in Hong Kong that adopts a stratified multistage cluster sampling design as in previous epidemiological studies (Lam et al., 2015). Young people between the ages of 15 and 24 at the time of recruitment were invited via mail sent to addresses obtained from the local government, which were randomly selected and clustered by districts and housing types. All assessments were conducted through face-to-face interviews, with the option of online video conferencing using the same procedures during COVID-19. Domains including suicide-related outcomes, psychiatric symptoms and disorders, personality and psychological factors, external stress exposure, and sociodemographic characteristics were assessed in this study. During the data collection period, it was estimated that our invitation letters were able to reach 5035 valid addresses (invalid addresses were defined as ‘age range not met’, ‘unit not occupied’, or ‘false address’). Of these valid addresses, 2956 participants (58.7%) were recruited, among which 2540 (85.9%) provided complete data on the variables of interest and were included in this study.

Seven participants (0.3%) of this sample reported being neither female nor male. Due to the small sample size, main analyses of this study focused on the biological sex of participants. Proportions of young people with suicidal ideation and behaviours in the past 12 months across each identified gender group are provided in the online Supplementary Material Table S1.

### Measures

#### Suicidal ideation, plan, and attempt

Lifetime and 12-month suicidal ideation, plan, and attempt, respectively, were assessed using items adapted from the Columbia–Suicide Severity Rating Scale (C-SSRS; Posner et al., 2011) as in previous large-scale suicide research (e.g. Nock *et al*. 2014, 2018). Suicidal ideation was assessed using the items ‘Did you ever in your life wish you were dead or would go to sleep and never wake up?’ or ‘Did you ever in your life have thought of killing yourself?’. Those who reported ‘yes’ to either of these items were further asked if they have ever made a suicide plan [‘Did you ever think about how you might kill yourself (e.g. taking pills, shooting yourself) or work out a plan of how to kill yourself?’] and suicide attempt [‘Have you ever made a suicide attempt (i.e. purposefully hurt yourself with at least some intent to die)?’]. The scale has shown good validity for use in young people (Posner et al., 2011).

#### Suicide-related rumination

Among those with lifetime suicidal ideation, participants were asked to rate the degree to which they have *ruminated* about their suicidal thoughts using a modified item also from the C-SSRS (Posner et al., 2011) as in Nock et al. (2018): ‘During that worst week, how easy was it for you to control those thoughts or push them out of your mind when you wanted to?’ on a 5-point Likert scale (‘easy’ to ‘impossible, unable to control the thoughts’).

#### Population-level and personal stressful events

Three types of external stressful events – social unrest-related TEs, COVID-19 PEs, and personal SLEs – were assessed. First, exposure to TEs since June 2019 was assessed using a three-item binary checklist capturing key experiences during the social unrest as has been previously reported (Wong et al., 2021b), including ‘crowd dispersal by the use of force’, ‘arrest or detention’, and ‘media viewing of others being physically attacked’.

COVID-19 PEs were asked using four items capturing stressful experiences related to COVID-19, including ‘having sufficient protective gears’ (reversed coded), ‘increased personal and rest time due to remote work/school’ (reversed coded), ‘increased conflicts with family due to remote work/school’, and ‘increased work/studies hours due to remote work/school’. Each item was assessed using a 5-point Likert scale (‘completely disagree’ to ‘completely agree’) before November 2020 and in the form of a binary checklist from November 2020. For those who completed their assessments before November 2020, a rating of ‘completely disagree’ or ‘disagree’ for the items ‘having sufficient gears’ and ‘personal and rest time increased’ and a rating of ‘agree’ or ‘completely agree’ for the other two items were recoded into 1. A score of 0 was given to those whose data were collected prior to the first confirmed case in Hong Kong (23 January 2020).

Personal SLEs were assessed using an adapted version of the List of Threatening Experiences (Brugha, Bebbington, Tennant, & Hurry, 1985), which is a 12-item binary checklist that captures different personal life stressors during the past year, such as serious illness, death of a first-degree or second-degree relative, and loss of a steady relationship (Brugha et al., 1985; Rosmalen, Bos, & de Jonge, 2012). ‘Expelled from school’ and ‘dropped out of school’ were provided as alternatives to ‘sacked from job’ and ‘unemployment’, respectively, to adapt to the current youth population. An ‘others’ option was also added, yielding a final number of 13 SLEs. The items endorsed in each of the measures were independently summed to generate composite scores for TEs, PEs, and SLEs, respectively.

#### Hopelessness and neuroticism

Hopelessness during the past week was assessed using the 20-item Beck's Hopelessness Inventory (Beck, Weissman, Lester, & Trexler, 1974). Items were rated on a binary checklist (true/false) and summed to generate a composite score. Neuroticism was assessed using the neuroticism subscale (eight items) of the 44-item Big Five Inventory (John & Srivastava, 1999). Items were rated on a 5-point Likert scale (‘disagree strongly’ to ‘agree strongly’) and summed to generate a composite score (*α* = 0.81).

#### Family functioning

Family functioning was assessed using the 16-item Brief Family Relationship Scale (Fok, Allen, & Henry, 2014), which captures three key domains, including family cohesion (e.g. ‘help and support each other’), expressiveness (e.g. ‘can talk openly in our home’), and conflict (e.g. ‘we argue a lot’), with each rated on a 4-point Likert scale (‘a lot’ to ‘not at all’). A higher score suggests poorer family functioning (*α* = 0.90).

#### Cognitive ability

Working memory, as reflective of cognitive ability, was assessed using the Forward and Backward Digit Span Test (DS-F and DS-B, respectively) in the Wechsler Adult Intelligence Scale, third edition (Wechsler, 1997). The DS-F requires participants to store the sequence of digits in their memory (more reflective of short-term memory), while the DS-B requires participants to process and reorganise information while storing the sequence of digits simultaneously (Grégoire & Van der Linden, 1997). A lower score on either of the tests suggests poorer working memory capability.

#### Non-suicidal self-harm, depressive disorder, and alcohol use

Lifetime non-suicidal self-harm was assessed using the item ‘Did you ever do something to hurt yourself on purpose, without wanting to die (e.g. cutting yourself, hitting yourself, or burning yourself)?’ from the Self-Injurious Thoughts and Behaviors Interview (Nock, Holmberg, Photos, & Michel, 2007). Any major depressive episode (MDE) during the past 12 months based on the DSM-V criteria was determined using items from the Composite International Diagnostic Interview–Screening Scales (Kessler et al., 2013). The degree of alcohol use was assessed using the 10-item Alcohol Use Disorders Identification Test (Saunders, Aasland, Babor, de la Fuente, & Grant, 1993), which captures aspects of hazardous alcohol use, dependence symptoms, and harmful use of alcohol, with each item rated on a 5-point Likert scale. A higher total score reflects higher risks in excessive alcohol use (*α* = 0.80).

#### Childhood adversity

Past adversity through the age of 17 was assessed using items adapted from the Adverse Childhood Experiences International Questionnaire (World Health Organization, 2018) as in previous studies (e.g. Mall et al., 2018), capturing aspects including abuse (physical, emotional, sexual), neglect, and parental psychopathology using a 5-point Likert scale (‘never’ to ‘very often’). Those who gave a rating of 3 (sometimes) to any of the experiences were categorised into a group denoting participants ‘with experience of childhood adversity’.

### Statistical methods

The prevalence of lifetime and 12-month suicidal ideation, ideation-only, plan, and attempt were each estimated with weighting adjustments according to age and sex data from the 2019 Hong Kong Census to improve representativeness.

Four separate multivariable logistic regression models were then applied to four key suicidal outcomes to examine their associated factors, namely 12-month suicidal ideation, ideation-only (i.e. excluding those without suicide plan or attempt), suicide plan, and suicide attempt. The variables included in each model included suicide-related rumination; exposure to TEs, PEs, and SLEs; hopelessness and neuroticism; family functioning; working memory (forward and backward digit span scores); lifetime non-suicidal self-harm (yes/no), 12-month MDE (yes/no), and 12-month alcohol use, and age, sex (female/male), and childhood adversity (yes/no). Online Supplementary analyses were performed to identify factors associated with 12-month ideation-only, plan, and attempt with age as a categorical variable (15–18/19–25 years) (see online Supplementary Material Table S3). Additionally, a two-stage design was applied to examine factors associated with 12-month suicide plan and attempt, respectively, among participants *with* suicidal ideation during the past 12 months. Adjusted odds ratios with 95% confidence intervals were reported, with statistical significance set at the *p* < 0.05 level. All analyses were performed using SPSS version 26.0.

## Results

### Prevalence of lifetime and 12-month suicidal ideation, plan, and attempt, suicide-related rumination, and non-suicidal self-harm among young people in Hong Kong

**Fig. 1.**
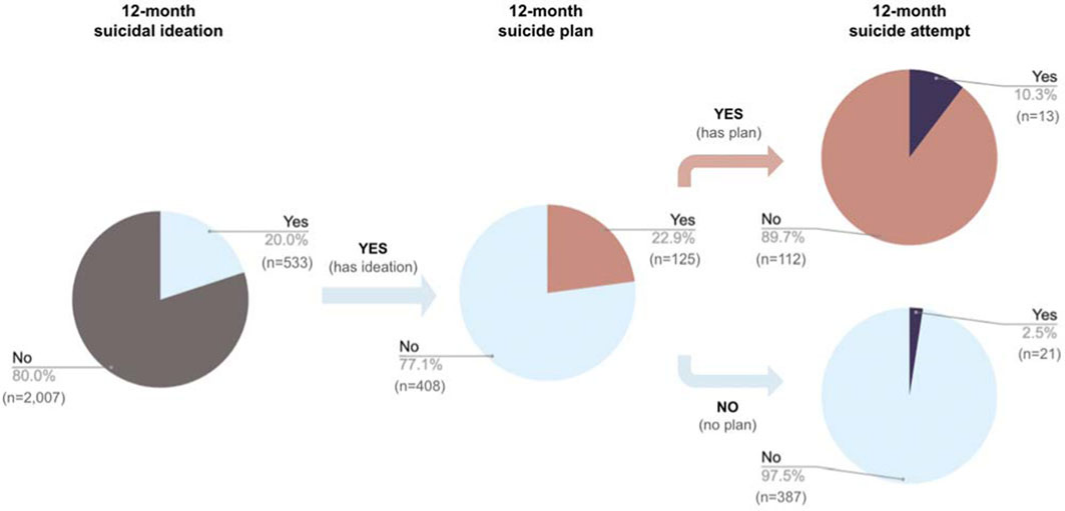
Flowchart illustrating the proportions of young people from the local epidemiological sample with 12-month suicide plan and attempt among those with suicidal ideation in Hong Kong. *Note.* Data are based on 2540 young people from the large epidemiological study in Hong Kong. Prevalence (%) is weighted according to age and sex data from the 2019 Hong Kong Census, with sample size (*n*) being unweighted.

Sample characteristics are presented in Table 1. After weighting adjustments, the lifetime prevalence of suicidal ideation, plan, and attempt in the Hong Kong youth population was 47.4, 14.6, and 5.5%, respectively. The weighted 12-month prevalence of suicidal ideation, plan, and attempt was 20.0, 4.6, and 1.3%, respectively. Suicidal ideation-only was reported in 15.4% of the local youth population (after weighting). A flowchart illustrating the 12-month prevalence of suicide plan and attempt among those with suicidal ideation are presented in Fig. 1.

**Table 1.** Multivariable logistic regression models showing the associations between the range of intrinsic and extrinsic factors and 12-month suicidal ideation, ideation-only, suicide plan, and suicide attempt in the representative epidemiological youth sample in Hong Kong (*n* = 2540)

	Overall sample (*n* = 2540)	12-month suicidal ideation (*n* = 533)				12-month suicidal ideation-only (no plan or attempt) (*n* = 387)				12-month suicide plan (*n* = 125)				12-month suicide attempt (*n* = 34)			
		OR	95% CI		*p*	OR	95% CI		*p*	OR	95% CI		*p*	OR	95% CI		*p*
Suicide-related rumination																	
Suicide-related rumination	1.06 (1.43)	**1.71**	**1.58**	**−1.86**	**<0.001**	**1.64**	**1.51**	**−1.79**	**<0.001**	**1.55**	**1.35**	**−1.77**	**<0.001**	**1.52**	**1.18**	**−1.95**	**0.001**
Extrinsic stressful events																	
Social unrest-related TEs	0.93 (0.71)	1.01	0.86	−1.19	0.91	1.02	0.85	−1.23	0.80	0.97	0.73	−1.29	0.83	1.20	0.74	−1.95	0.47
COVID-19 PEs	1.49 (0.96)	0.90	0.79	−1.02	0.095	0.88	0.77	−1.01	0.068	0.97	0.78	1.20	0.75	**1.61**	**1.07**	**−2.41**	**0.021**
Personal SLEs	0.82 (1.12)	0.98	0.89	−1.08	0.70	0.94	0.84	−1.05	0.25	1.09	0.93	−1.27	0.31	1.23	0.95	−1.59	0.11
Psychological and personality																	
Hopelessness	6.84 (4.08)	**1.09**	**1.06**	**−1.13**	**<0.001**	**1.08**	**1.05**	**−1.12**	**<0.001**	**1.11**	**1.05**	**−1.17**	**<0.001**	1.02	0.93	−1.12	0.72
Neuroticism	25.68 (5.67)	**1.07**	**1.05**	**−1.10**	**<0.001**	**1.08**	**1.05**	**−1.11**	**<0.001**	1.04	1.00	−1.09	0.076	1.00	0.92	−1.09	0.97
Interpersonal factors																	
Poorer family functioning	19.87 (7.16)	**1.04**	**1.02**	**−1.06**	**<0.001**	**1.04**	**1.02**	**−1.06**	**<0.001**	1.03	1.00	−1.06	0.098	**1.08**	**1.02**	**−1.13**	**0.006**
Cognitive ability																	
Forward digit span	12.71 (1.44)	0.93	0.86	−1.01	0.088	0.98	0.89	−1.07	0.60	**0.82**	**0.72**	**−0.94**	**0.004**	0.91	0.73	−1.15	0.44
Backward digit span	9.75 (2.97)	1.02	0.98	−1.06	0.43	1.01	0.96	−1.05	0.75	1.05	0.98	−1.13	0.19	0.99	0.87	−1.12	0.83
Self-harm and psychiatric condition																	
No lifetime non-suicidal self-harm, *n* (%)	2160 (85)	Ref				Ref				Ref				Ref			
Has lifetime non-suicidal self-harm, *n* (%)	380 (15)	1.33	0.99	−1.79	0.056	1.16	0.83	−1.60	0.39	**1.59**	**1.01**	**−2.49**	**0.045**	**4.03**	**1.71**	**−9.48**	**0.001**
No 12-month MDE, *n* (%)	2159 (85)	Ref				Ref				Ref				Ref			
Has 12-month MDE, *n* (%)	381 (15)	**1.90**	**1.42**	**−2.53**	**<0.001**	**1.60**	**1.16**	**−2.21**	**0.004**	**2.73**	**1.75**	**−4.24**	**<0.001**	1.99	0.87	−4.54	0.10
Alcohol use	2.12 (3.36)	1.00	0.97	−1.04	0.90	1.00	0.96	−1.04	0.91	1.01	0.95	−1.06	0.86	1.02	0.93	−1.11	0.74
Demographics and background																	
Age	19.84 (2.78)	**0.93**	**0.89**	**−0.97**	**0.001**	**0.93**	**0.88**	**−0.97**	**0.001**	0.96	0.89	−1.04	0.34	0.98	0.85	−1.13	0.79
Male, *n* (%)	1062 (41.8)																
Female, *n* (%)	1478 (58.2)	**1.39**	**1.09**	**−1.78**	**0.009**	1.29	0.99	−1.69	0.056	**1.66**	**1.04**	**−2.64**	**0.034**	1.23	0.51	−2.96	0.65
No childhood adversity, *n* (%)	1612 (63.5)																
Has childhood adversity, *n* (%)	928 (36.5)	0.95	0.74	−1.23	0.71	0.97	0.74	−1.29	0.85	1.03	0.65	−1.64	0.90	0.66	0.28	−1.54	0.34

MDE, major depressive episode; PEs, COVID-19 pandemic-related events; SLEs, personal stressful life events; TEs, social unrest-related traumatic events.

*Note.* Statistics are presented in the form of mean (s.d.), unless stated otherwise. Values significant at the *p* < 0.05 level are in boldface.

Regarding suicide-related rumination, 5.8 and 3.4% of the participants (after weighting) reported the thoughts being ‘very difficult’ and ‘impossible’ to control even when they wanted to, respectively. Meanwhile, the weighted prevalence of lifetime non-suicidal self-harm was 14.0%.

### Factors associated with 12-month suicidal ideation, ideation-only, plan, and attempt in the epidemiological youth sample

Table 1 presents findings from the four respective multivariable logistic regression models for each of the 12-month suicidal outcomes. Some similarities and differences in the patterns of associations were observed. Summarised findings from the regression models are presented in online Supplementary Material Table S2 for ease of comparison.

#### Shared factors associated with 12-month suicidal outcomes

Among the variables, suicide-related rumination was among the only factor that was significantly associated with all four suicide-related outcomes, including suicidal ideation (OR 1.71, CI 1.58–1.86), ideation-only (OR 1.64, CI 1.51–1.79), plan (OR 1.55, CI 1.35–1.77), and attempt (OR 1.52, CI 1.18–1.95) (all *p* < 0.01).

Meanwhile, poorer family functioning was associated with elevated odds of 12-month suicidal ideation and ideation-only (OR 1.04, CI 1.02–1.06 for both) and attempt (OR 1.08 CI 1.02–1.13), but not with plan. Higher levels of hopelessness and having an MDE in the past 12 months were associated with elevated odds of suicidal ideation, ideation-only, and plan (all *p* < 0.05), but not with attempt. Female sex was associated with both suicidal ideation and plan (both *p* < 0.05). Meanwhile, self-harm was associated with suicide plan and attempt (both *p* < 0.05), but not with ideation (Table 1).

#### Unique contributing factors to 12-month suicidal outcomes

Apart from the shared risk factors, a number of other factors showed specificity in their associations with the respective suicide-related outcomes. Personal background factors, such as higher degrees of neuroticism and younger age, were associated with elevated odds of suicidal ideation (OR 1.07, CI 1.05–1.10 and OR 0.93, CI 0.89–0.97, respectively) and ideation-only (OR 1.08, CI 1.05–1.11 and OR 0.93, CI 0.88–0.97, but not with plan or attempt (Table 1). Poorer working memory capacity, as reflected by forward digit span score, was associated specifically with suicide plan (OR 0.82, CI 0.72–0.94). Meanwhile, COVID-19 PEs were specifically associated with elevated odds of suicide attempt (OR 1.61, CI 1.07–2.41).

### Factors associated with 12-month suicide plan and attempt among those with ideation: a two-stage approach

Further analyses were performed to determine factors associated with 12-month suicide plan and attempt among young people with suicidal ideation in the past 12 months (*n* = 533).

Suicide-related rumination remained to be significantly associated with elevated odds of 12-month suicide plan (OR 1.24, CI 1.05–1.47) (Table 2). A non-significant positive trend was observed between suicide-related rumination and suicide attempt (OR 1.32, CI 0.98–1.78, *p* = 0.064). Poorer working memory capacity (OR 0.84, CI 0.73–0.97) and 12-month MDE (OR 2.04, CI 1.27–3.28) were specifically associated with suicide plan. Meanwhile, with all other factors accounted for, COVID-19 PEs (OR 1.72, CI 1.13–2.62), poorer family functioning (OR 1.06, CI 1.01–1.12), self-harm (OR 3.60, 1.56–8.33), as well as personal SLEs (OR 1.32, CI 1.00–1.74), were among specifically associated with elevated odds of suicide attempt (Table 2).

**Table 2.** Factors associated with 12-month suicide plan and 12-month suicide attempt among those with suicidal ideation during the past 12 months (*n* = 533)

Among those with 12-month suicidal ideation (*n* = 533)	12-month suicide plan (*n* = 125)				12-month suicide attempt (*n* = 34)			
	OR	95% CI		*p*	OR	95% CI		*p*
Suicide-related rumination								
Suicide-related rumination	**1**.**24**	**1**.**05**	**−1.47**	**0**.**014**	1.32	0.98	−1.78	0.064
External stressful events								
Social unrest-related TEs	0.96	0.71	−1.28	0.77	1.26	0.76	−2.08	0.38
COVID-19 PEs	1.01	0.80	−1.29	0.92	**1**.**72**	**1**.**13**	**−2.62**	**0**.**011**
Personal SLEs	1.13	0.95	−1.35	0.16	**1**.**32**	**1**.**00**	**−1.74**	**0**.**047**
Psychological and personality								
Hopelessness	1.06	1.00	−1.12	0.055	0.96	0.86	−1.06	0.37
Neuroticism	1.01	0.96	−1.06	0.82	0.98	0.89	−1.07	0.58
Interpersonal factors								
Poorer family functioning	1.00	0.97	−1.04	0.88	**1**.**06**	**1**.**01**	**−1.12**	**0**.**031**
Cognitive ability								
Forward digit span	**0**.**84**	**0**.**73**	**−0.97**	**0.016**	0.96	0.76	–1.20	0.70
Backward digit span	1.07	0.99	−1.16	0.11	1.00	0.87	−1.15	0.96
Self-harm and psychiatric condition								
No lifetime self-harm attempt, *n* (%)	Ref				Ref			
Has lifetime self-harm attempt, *n* (%)	1.49	0.94	−2.38	0.091	**3**.**60**	**1**.**56**	**−8.33**	**0.003**
No 12-month MDE, *n* (%)	Ref				Ref			
Has 12-month MDE, *n* (%)	**2**.**04**	**1**.**27**	**−3.28**	**0.003**	1.33	0.58	−3.09	0.50
Alcohol use	1.00	0.94	−1.06	0.99	1.02	0.94	−1.11	0.61
Background factors								
Age	1.02	0.94	−1.10	0.70	1.01	0.88	−1.17	0.86
Male	Ref				Ref			
Female	1.49	0.91	−2.45	0.12	1.18	0.48	−2.87	0.72
No childhood adversity	Ref				Ref			
Has childhood adversity	1.02	0.63	−1.66	0.93	0.61	0.26	−1.45	0.26

MDE, major depressive episode; PEs, COVID-19 pandemic-related events; SLEs, personal stressful life events; TEs, social unrest-related traumatic events.

*Note*. Values are presented in the form of mean (s.d.), unless stated otherwise.

## Discussion

Using data from a representative epidemiological sample of young people in Hong Kong, we found nearly half of the local youth population (47.3%) have had suicidal thoughts at least once or more in their lifetime, one in seven (14.6%) have made a plan, and one in 18 (5.5%) have ever made an attempt. The prevalence of 12-month suicidal ideation in the Hong Kong youth population was also as high as 20.0%, which is slightly higher than those reported in a previous local study among secondary school students (13.7% in Shek and Yu, 2012) and in other meta-analysis studies (18.8% among high school students in the United States between 1991 and 2017 in Lindsey, Sheftall, Xiao, and Joe, 2019; 18.7% among left-behind children in China in Qu et al., 2021).

Importantly, our findings showed that suicide-related rumination was among the only factor associated not only with suicidal ideation but also plan and attempt. This observation extended findings from a number of recent works (Rogers & Joiner, 2018; Rogers, Gallyer, et al., 2021; Rogers, Law, et al., 2021) to suggest its utility in identifying young people at greater suicidal risks. Notably, we provided novel evidence to show the specific associations between COVID-19 stressors and suicide attempt beyond other previously reported intrinsic and personal-level extrinsic risk factors of suicide. The importance of accounting for the influences of suicide-specific intrinsic factors, as well as both personal and population-level extrinsic factors, to guide more accurate and effective suicide prevention and intervention work is highlighted.

### Significance of suicide-related rumination

Ruminative experiences related to suicidal thoughts may reflect a form of preservative cognition in which one's attention is disproportionately narrowed to suicide-related content (Whitmer & Gotlib, 2013). This would be in line with previous findings in suggesting attentional biases to and fixating on suicide-related contents could act as cognitive warning signs for suicidal behaviours (Cha, Najmi, Park, Finn, & Nock, 2010; Moscardini, Aboussouan, Bryan, & Tucker, 2020). The notion of ‘cognitive rigidity’ in the cognitive model of suicide (Ellis & Rutherford, 2008) may also be relevant. Notably, suicide-related rumination not only may be modifiable (Grierson, Hickie, Naismith, & Scott, 2016; Holdaway, Luebbe, & Becker, 2018; Watkins et al., 2011), but could also be understood as increased ‘stickiness’ of suicidal thoughts in one's mental representation (Joormann, Levens, & Gotlib, 2011), which may help to reduce the stigma associated with suicidal behaviours.

### Significance of non-suicidal self-harm

The finding that non-suicidal self-harm was a relatively robust risk factor for suicide attempt is consistent with the literature (Castellví et al., 2017; O'Connor et al., 2018). Thoughts about self-harm have also been suggested to indicate risks for psychiatric conditions and have long-term consequences on physical health and functioning (Bjureberg et al., 2019; Turner, Chapman, & Layden, 2012). As with the tendency to conceal or deny suicidal behaviours, nonetheless, only a minority of young people would disclose their self-harm experiences and seek help due to the fear of being perceived as ‘attention-seeking’ (Michelmore & Hindley, 2012; Rowe et al., 2014). Considering other indicators that are less stigmatising (e.g. ever had suicide-related rumination, poor family functioning, exposure to population-level stressors) would thus also be important to confirm the level of risk among those who did and did not report no self-harm experience.

### Significance of personal and population-level external stressors

The observations that COVID-19 stressful events and poorer family functioning showed stronger associations with suicide attempt, rather than intrinsic vulnerabilities, are thus critical. As the common perceptions of suicidal behaviours tended to emphasise individual vulnerabilities (Batterham, Calear, & Christensen, 2013; Carpiniello & Pinna, 2017), placing greater weight on environmental influences may further help to reduce stigma and facilitate help-seeking. Importantly, in the case of not only Hong Kong but also many other societies, the effect of COVID-19 on suicidal behaviours could have been exacerbated by other prior or concurrent population-level stressors, such as social protests, financial crises, and environmental crises (Hou et al., 2021; Moutier, 2021; Wong et al., 2021c). The resulting societal, lifestyle, and psychosocial changes can make meaning-making and adaptations difficult in young people, who are simultaneously undergoing critical developmental transitions (Arain et al., 2013; Park, 2010). The role of the family in suicidal outcomes during youth may be even more pronounced in collectivistic cultures like Hong Kong, where family relationships are highly valued (Ho et al., 2016). Supporting parents or guardians in cultivating more open communications may help young people better cope with these circumstances and facilitate early risk detection and help-seeking.

### Identifying those at greater suicidal risk using a two-stage approach

To further facilitate the identification of young people at greater suicidal risks in population and clinical settings, a two-stage design can be useful (i.e. selecting those with suicidal ideation). Based on our analyses, suicide-related rumination appeared to remain a strong indicator of suicidal risk (significant for suicide plan and positive trend for attempt). Interestingly, whereas other intrinsic factors (poorer cognitive ability and history of depressive disorder) showed stronger associations with suicide plan, the experience of external triggers (COVID-19 stressors, poorer family functioning, and personal SLEs), as well as behavioural indicators (lifetime non-suicidal self-harm), may play more critical roles in suicide attempt. These observations shed light on the potential interplay of intrinsic-extrinsic factors across the various suicidal outcomes, which have implications both on our understanding of suicidality and intervention design.

Notably, during the period of social unrest and COVID-19, the disruptions to interpersonal relationships, education, and employment could have added to the cognitive load of young people. Suicide and ‘self-sacrifice’ were indeed common sentiments among young people on online platforms at the time (Yip & Pinkney, 2022). Young people with fewer cognitive resources (as reflected in general intellectual levels) could possibly be more susceptible to such influences (Bittár, Falkstedt, & Wallin, 2020). Coupled with difficulties in coping and identifying alternative solutions (Bredemeier & Miller, 2015), suicide may be viewed as the ‘only option’ (Na et al., 2020). The additional experiences of significant external stressors can possibly cause a shift to the ‘already fragile’ system and lead it to move past a ‘tipping point’ (de Beurs et al., 2021; Kalisch et al., 2019), resulting in suicide attempts.

### Strengths and limitations

To the best of our knowledge, this was to date the first youth-specific epidemiological study on suicidal behaviours conducted in Hong Kong and among the first to be conducted during COVID-19 globally. Apart from presenting an update on the local prevalence of youth suicidal behaviours, the current study also offered insights into the markers and risk factors associated with youth suicidal ideation, plan, and attempt, respectively. In the hope to improve future suicide screening in the population and clinical decisions, a two-stage approach was further applied to identify factors associated with more severe suicidal behaviours (plan and attempt) among those at increased risk (with suicidal ideation). The differences in risk factors and potential underlying mechanisms across these suicidal outcomes are also suggested.

Some limitations of this study were nonetheless acknowledged. First, the cross-sectional nature of this study prevented the determination of the direction of associations among the putative factors and outcomes. For instance, while memory impairments may prevent appropriate retrieval of past experiences for identifying solutions to current issues, which contributes to risk for suicide plan (Richard-Devantoy, Berlim, & Jollant, 2015), it is also possible that prolonged engagement in suicide planning and continued ruminations could further deplete cognitive resources (Whitmer & Gotlib, 2013). It would be helpful for future studies to adopt a longitudinal study design to test our study findings.

Furthermore, while some of our observations were in line with previous findings, some differences were also noted. For instance, we did not find excessive drinking to be associated with suicidal outcomes as in previous overseas studies (Darvishi, Farhadi, Haghtalab, & Poorolajal, 2015). One reason may owe to the comparatively lower binge-drinking rate among young people in Hong Kong (Huang, Ho, Wang, Lo, & Lam, 2016; Rotheray et al., 2011). We also did not find gender minority to be associated with elevated suicidal risks (Horwitz et al., 2020; Jackman, Caceres, Kreuze, & Bockting, 2021), plausibly due to the small proportion of participants identifying as non-cis-gender in our epidemiological sample (*n* = 7; 0.3%). Nonetheless, early detection and intervention work specifically targeted at this group may be helpful to best support these young individuals. Greater attention should also be given to this population in future suicide-related research.

We also note that the associated odds in our multivariable models were generally small, which may partly relate to the use of a population-based epidemiological rather than clinical sample. Indeed, the values were similar to those reported in a previous large community-based study (Fairweather, Anstey, Rodgers, & Butterworth, 2006). The wide-ranging factors we accounted for, as opposed to a limited set of factors, might also have contributed to such differences. It would be worthwhile for future studies to apply a similar model to test the patterns of associations across cultures and in different populations and settings.

Finally, while the single-item measure of suicide-related rumination can be beneficial in low-resource and time-limited settings and in engaging young people (Wong et al., 2021d), a more detailed assessment would be helpful to improve our understanding of this phenomenon. Capturing the daily moment-to-moment experiences of suicide-related thoughts and rumination through the experience sampling method may also help to establish a more fine-grained picture of these experiences and determine their associations with momentary mood states in real-world contexts (Kleiman & Nock, 2018; Myin-Germeys et al., 2018).

## Conclusion

In the current global context of ongoing stress, preparing clinicians and other service providers for the early detection of young people at heightened risk for suicidal behaviours is ever more important. Suicide-related rumination revealed to be a promising marker that is not only modifiable but also less stigmatising. Efforts in conveying the message about the significance of not just personal but also other circumstantial and population-level influences in suicidal outcomes to the public can also be critical in reducing stigma and improving help-seeking. Developing strategies for reducing suicide-related rumination, mitigating the impact of COVID-19 stresses, and improving family functioning can be important in future suicide prevention work.

## Supplementary material

For supplementary material accompanying this paper visit https://doi.org/10.1017/S0033291722001519. click here to view supplementary material
