# Supplementary material for: Prevalence and correlates of suicidal behaviours in a representative epidemiological youth sample in Hong Kong: the significance of suicide-related rumination, family functioning, and ongoing population-level stressors
Source: Psychol Med. 2022 Jun 2;53(10):4603–13. doi: 10.1017/S0033291722001519 (PMC10388322; doi:10.1017/S0033291722001519)
Supplement: Supplementary file 1 [file S0033291722001519sup001.pdf]

Supplementary Material

Prevalence and correlates of suicidal behaviours in a representative epidemiological youth sample in Hong Kong: the significance of suicide-related rumination, family functioning, and ongoing population-level stressors

Stephanie MY Wong[1#], Charlie H Ip[1#], Christy LM Hui[1], YN Suen[1], Corine SM Wong[1], WC Chang[1,2], Sherry KW Chan[1,2], Edwin HM Lee[1], Simon SY Lui[1], KT Chan[1], Michael TH Wong[1], Eric YH Chen[1,2]*

*Corresponding author
[#]Equal contribution

[1]Department of Psychiatry, School of Clinical Medicine, LKS Faculty of Medicine, The University of Hong Kong, Hong Kong
[2]The State Key Laboratory of Brain and Cognitive Sciences, The University of Hong Kong, Hong Kong

Corresponding author
Eric Y. H. Chen
MA(Oxon), MBChB(Edin), MD(Edin), FRCPsych, FHKAM(Psychiatry)
Chair Professor, Department of Psychiatry, University of Hong Kong

Address: Department of Psychiatry, University of Hong Kong, 2/F New Clinical Building,
Queen Mary Hospital, Pokfulam Road, Hong Kong
Email: eyhchen.hk@gmail.com
Tel: (852) 2255 4488

Table S1. Proportions of young people from the representative epidemiological sample in Hong Kong with 12-month suicidal behaviours across each gender group (n = 2,540)

Gender	Overall sample (n = 2,540)	12-month suicidal ideation (n = 533)	12-month suicidal ideation-only (n = 387)	12-month suicide plan (n = 125)	12-month suicide attempt (n = 34)
Male	1,059 (41.7)	168 (31.5)	132 (34.1)	30 (24.0)	Male 8 (23.5)
Female	1,474 (58.0)	363 (68.1)	254 (65.6)	94 (75.2)	26 (76.5)
Asexual	1 (0.0)	–	–	–	–
Transgender	2 (0.1)	1 (0.2)	1 (0.3)	–	–
Non-binary	3 (0.1)	1 (0.2)	–	1 (0.8)	–
Other	1 (0.0)	–	–	–	–

Note. Sample sizes and proportions are unweighted.

Table S2. Summarised findings from the multivariable logistic regression models for 12-month suicidal ideation, ideation-only, plan, and attempt in the representative epidemiological youth sample in Hong Kong (n = 2,540)

**Construct**		**12-month suicidal ideation** (n = 533)	**12-month suicidal ideation-only** (n = 387)	**12-month suicide plan** (n = 125)	**12-month suicide attempt** (n = 34)
**Suicide-related rumination**	Suicide-related rumination	✓	✓	✓	✓
**Extrinsic stressful events**	Social unrest-related TEs	n.s.	n.s.	n.s.	n.s.
	COVID-19 PEs	n.s.	n.s.	n.s.	✓
	Personal SLEs	n.s.	n.s.	n.s.	✓
**Personality and psychological factors**	Hopelessness	✓	✓	✓	n.s.
	Neuroticism	✓	✓	n.s.	n.s.
**Interpersonal factors**	Family conflicts	✓	✓	n.s.	✓
**Cognitive ability**	Forward digit span	n.s.	n.s.	✓	n.s.
	Backward digit span	n.s.	n.s.	n.s.	n.s.
**Experience of self-harm and psychiatric condition**	Lifetime non-suicidal self-harm	n.s.	n.s.	✓	✓
	12-month MDE	✓	✓	✓	n.s.
	Excessive alcohol use	n.s.	n.s.	n.s.	n.s.
**Background factors**	Younger age	✓	✓	n.s.	n.s.
	Female	✓	n.s.	✓	n.s.
	Childhood adversity	n.s.	n.s.	n.s.	n.s.

Note. n.s. signifies non-significant findings from the separate multivariable logistic regression models. MDE = major depressive episode; PEs = COVID-19 pandemic-related events; SLEs = personal stressful life events; TEs = social unrest-related traumatic events.

Table S3. Multivariable logistic regression models showing the associations between the range of intrinsic and extrinsic factors and 12-month suicidal ideation, ideation-only, suicide plan, and suicide attempt in the representative epidemiological youth sample with age as a categorical variable

	Overall sample (n = 2,540)	12-month suicidal ideation (n = 533)			12-month suicidal ideation-only (no plan or attempt) (n = 387)			12-month suicide plan (n = 125)			12-month suicide attempt (n = 34)		
		OR	95% CI	*p*	OR	95% CI	*p*	OR	95% CI	*p*	OR	95% CI	*p*
Suicide-related rumination													
Suicide-related rumination	1.06 (1.43)	**1.71**	**1.58 – 1.85**	**<0.001**	**1.64**	**1.51 – 1.79**	**<0.001**	**1.55**	**1.35 – 1.77**	**<0.001**	**1.52**	**1.18 – 1.95**	**0.001**
Extrinsic stressful events													
Social unrest-related TEs	0.93 (0.71)	1.00	0.85 – 1.18	1.00	1.01	0.85 – 1.21	0.89	0.97	0.73 – 1.28	0.81	1.20	0.74 – 1.95	0.46
COVID-19 PEs	1.49 (0.96)	0.90	0.79 – 1.02	0.088	0.88	0.77 – 1.01	0.066	0.97	0.78 – 1.20	0.76	**1.60**	**1.07 – 2.40**	**0.021**
Personal SLEs	0.82 (1.12)	0.98	0.89 – 1.08	0.70	0.94	0.84 – 1.05	0.25	1.09	0.93 – 1.27	0.31	1.23	0.96 – 1.60	0.11
Psychological and personality													
Hopelessness	6.84 (4.08)	**1.09**	**1.06 – 1.13**	**<0.001**	**1.08**	**1.05 – 1.12**	**<0.001**	**1.11**	**1.05 – 1.17**	**<0.001**	1.02	0.93 – 1.12	0.74
Neuroticism	25.68 (5.67)	**1.07**	**1.05 – 1.10**	**<0.001**	**1.07**	**1.05 – 1.10**	**<0.001**	1.04	1.00 – 1.09	0.079	1.00	0.92 – 1.09	0.94
Interpersonal factors													
Poorer family functioning	19.87 (7.16)	**1.04**	**1.02 – 1.06**	**<0.001**	**1.04**	**1.02 – 1.06**	**<0.001**	1.03	1.00 – 1.06	0.10	**1.08**	**1.02 – 1.13**	**0.006**
Cognitive ability													
Forward digit span	12.71 (1.44)	0.93	0.85 – 1.01	0.079	0.97	0.89 – 1.07	0.58	**0.82**	**0.71 – 0.94**	**0.004**	0.92	0.73 – 1.15	0.45
Backward digit span	9.75 (2.97)	1.02	0.98 – 1.06	0.41	1.01	0.96 – 1.05	0.73	1.05	0.98 – 1.13	0.19	0.99	0.87 – 1.12	0.83
Self-harm and psychiatric condition													
No lifetime non-suicidal self-harm, n (%)	2,160 (85)	Ref			Ref			Ref			Ref		
Has lifetime non-suicidal self-harm, n (%)	380 (15)	**1.35**	**1.01 – 1.82**	**0.044**	1.17	0.84 – 1.63	0.34	**1.61**	**1.03 – 2.52**	**0.038**	**4.01**	**1.71 – 9.42**	**0.001**
No 12-month MDE, n (%)	2,159 (85)	Ref			Ref			Ref			Ref		
Has 12-month MDE, n (%)	381 (15)	**1.89**	**1.42 – 2.52**	**<0.001**	**1.59**	**1.15 – 2.19**	**0.005**	**2.73**	**1.75 – 4.25**	**<0.001**	2.00	0.87 – 4.56	0.10
Alcohol use	2.12 (3.36)	1.00	0.97 – 1.03	0.96	0.99	0.96 – 1.03	0.76	1.00	0.95 – 1.06	0.90	1.02	0.93 – 1.11	0.71
Demographics and background, n (%)													
Aged 19–25 years	1,682 (66.2)	Ref			Ref			Ref			Ref		
Aged 15–18 years	858 (33.8)	**1.35**	**1.05 – 1.73**	**0.018**	**1.35**	**1.04 – 1.76**	**0.027**	1.15	0.74 – 1.79	0.53	1.20	0.54 – 2.65	0.65
Male	1,062 (41.8)	Ref			Ref			Ref			Ref		
Female	1,478 (58.2)	**1.37**	**1.07 – 1.76**	**0.012**	1.28	0.98 – 1.66	0.071	**1.64**	**1.03 – 2.61**	**0.038**	1.22	0.51 – 2.95	0.65
No childhood adversity	1,612 (63.5)	Ref			Ref			Ref			Ref		
Has childhood adversity	928 (36.5)	0.95	0.73 – 1.23	0.69	0.97	0.73 – 1.28	0.81	1.03	0.65 – 1.63	0.91	0.66	0.28 – 1.55	0.34

Note. Values are presented in the form of mean (SD), unless stated otherwise. MDE = major depressive episode; PEs = COVID-19 pandemic-related events; SLEs = personal stressful life events; TEs = social unrest-related traumatic events.
